# Preoperative C-reactive protein to albumin ratio may be a good prognostic marker in patients undergoing hepatectomy for hepatocellular carcinoma: a meta-analysis

**DOI:** 10.3389/fnut.2024.1444352

**Published:** 2024-10-01

**Authors:** Shi Wang, Shengqian Xu, Jun Wang, Hailin Ye, Kai Zhang, Xiaopeng Fan, Xiaoya Xu

**Affiliations:** ^1^Lishui City People's Hospital, Lishui, China; ^2^Department of Critical Care Medicine, Second Affiliated Hospital, Zhejiang University School of Medicine, Hangzhou, China; ^3^Department of General Surgery, Lishui People’s Hospital, Lishui, China

**Keywords:** C-reactive protein to albumin ratio, prognostic marker, hepatocellular carcinoma, hepatectomy, meta-analysis

## Abstract

**Background:**

Systemic inflammatory response represented by C-reactive protein to albumin ratio (CAR) was shown to be associated with long-term outcome in patients with hepatocellular carcinoma (HCC). We conducted a meta-analysis to investigate the prognostic value of preoperative CAR in patients undergoing hepatectomy for HCC.

**Methods:**

We searched four databases (PubMed, Embase, Scopus and Cochrane Library) from inception to May 10th, 2024. Studies investigating the prognostic value of preoperative CAR in HCC patients after hepatectomy. The primary endpoints were overall survival (OS) and disease-free survival (DFS). Data from individual studies were aggregated to calculate the pooled hazard ratio (HR) using a random-effects model.

**Results:**

A total of 11 studies included 4,066 patients were finally analyzed in the meta-analysis. Overall, the higher preoperative CAR was associated with poorer OS (HR 1.92, 95% CI 1.67 to 2.22, *I*^2^ = 0%) and DFS (HR 1.79, 95% CI 1.59 to 2.02, *I*^2^ = 0%) rate. Furthermore, subgroup analyses indicated that CAR could be a prognostic biomarker for patients with HCC regardless of regions and cut-off value.

**Conclusion:**

Our meta-analysis indicates that higher preoperative CAR level is associated with poorer OS and DFS, it may be a good prognostic marker of survival outcomes after hepatectomy in patients with HCC. However, future prospective trials are necessary to validate the conclusion.

**Systematic review registration:**

The study protocol was registered in the Open Science Framework (https://osf.io/uavt8).

## Background

Despite advances in cancer treatment, liver cancer remains one of the most prevalent malignancies and constitutes a significant global public health challenge (1). According to the GLOBOCAN 2022, liver cancer ranks as the sixth most commonly diagnosed cancer and the third leading cause of cancer-related mortality, accounting for over 750,000 deaths worldwide in 2022 (2). Current treatment strategies available for liver cancer encompass surgery, chemotherapy, radiation, immunotherapies, or combinations thereof, with hepatectomy serves as the primary radical treatment for resectable liver cancer (3). However, a majority of patients experience high recurrence rates post-surgery, leading to suboptimal long-term survival outcomes (4). Consequently, the accurate identification of high-risk patients with poor prognoses through an efficient and convenient preoperative model is crucial. This identification facilitates the optimization of adjuvant therapy and potentially enhances long-term prognosis (5, 6).

Systemic inflammation within the tumor microenvironment is increasingly recognized as a crucial prognostic indicator in cancer patients (7). Serum C-reactive protein (CRP) and albumin (ALB), two significant acute phase-proteins, serve as markers reflecting the body’s nutritional status during a systemic inflammatory response (8). An increasing body of research has demonstrated their association with survival prognosis in cancer patients (9, 10). The CRP to ALB ratio (CAR) has recently emerged as a novel and potentially useful inflammation-based prognostic indicator for solid tumors. Recent studies have identified CAR as a reliable and effective prognostic marker for various cancers, including esophageal, gastric, pancreatic, colorectal, and nasopharyngeal cancers (11–15). Several recent studies have indicated that elevated CAR is associated with poorer survival rates and higher postoperative recurrence rates in patients with hepatocellular carcinoma (HCC) (16–19). Nevertheless, due to the heterogeneous biological behaviors of HCC, which can influence the clinical presentation of inflammation-related markers, the prognostic significance of CAR in assessing outcomes for HCC patients remains uncertain (20, 21). Therefore, we conducted this meta-analysis to investigate the prognostic value of CAR for overall survival (OS) and disease-free survival (DFS) in patients who have undergone hepatectomy for HCC.

## Methods

### Study selection

This meta-analysis was conducted in accordance with the updated PRISMA guidelines (22) (see Supplementary material 1). The study protocol was pre-registered in the Open Science Framework.^1^ Two authors independently conducted a comprehensive literature search in PubMed, Embase, Scopus, and Cochrane Library from their inception to May 10, 2024 for relevant articles published in English. The search strategy incorporated keywords such as “CRP,” “ALB,” “CAR,” and “liver cancer.” Detailed search strategies are provided in Supplementary material 2.

The inclusive criteria were as follows:

1. Adult patients undergoing hepatectomy for HCC, with HCC confirmed by postoperative pathology;
2. Evaluation of preoperative CRP and ALB levels in serum samples;
3. Reported clinical outcomes including OS and DFS;
4. Both prospective and retrospective studies, without design restrictions;
5. Studies published in English only.

The exclusion criteria were as follows:

1. Duplicate publications or studies involving heterogeneous patient types;
2. Case reports, non-human studies, studies lacking sufficient information or relevant outcomes, and studies focusing on special populations (e.g., pediatric, pregnant);
3. Studies including patients who did not undergo hepatectomy for HCC.

### Data extraction and quality assessment

The retrieval of relevant studies were conducted independently by two authors (Shi Wang, and Shengqian Xu). All potentially eligible studies underwent full-text screening for inclusion. Discrepancies in this process were resolved through discussion and consensus. In cases where consensus could not be reached, a third co-author (Xiaopeng Fan) arbitrated to resolve the issue. A study-specific data abstraction form was adapted from the standardized Cochrane Data Collection template. Two authors (Jun Wang and Hailin Ye) independently extracted data, including the first author, publication year, country, sample size, population characteristics, cutoff value, and follow-up period. Predefined outcomes from the included studies were similarly extracted. When data were unavailable in the published articles, the corresponding authors were contacted to obtain essential missing information.

The quality of included studies was independently assessed by two reviewers (Shi Wang, and Shengqian Xu) using the Newcastle-Ottawa Scale (23). Publication bias was evaluated using the Egger’s regression test. When publication bias was identified, the trim-and-fill method was employed to further assess its potential impact on our meta-analysis (24). Any discrepancies throughout all phases were ultimately resolved through team consensus.

### Statistical synthesis and analysis

We performed a pooled analysis to assess the relationship between CAR and OS and DFS in patients with HCC. A random-effects model was employed to estimate the hazard ratio (HR) along with a 95% confidence interval (CI). The I^2^ statistic was computed to gage heterogeneity between studies, where *I*^2^ values of <25%, 25 to 75, and > 75% signify low, moderate, and high heterogeneity, respectively (25). We stratified studies based on region and cutoff value (cutoff ≥0.02 versus ≥0.05) for subgroup analysis. Additionally, we conducted a sensitivity analysis to assess the impact of individual studies by omitting one at a time. All analyses were performed using the R software environment (version 4.3.1), with statistical significance set at *p* < 0.05.

## Results

### Study selection and study characteristics

**Figure 1:**
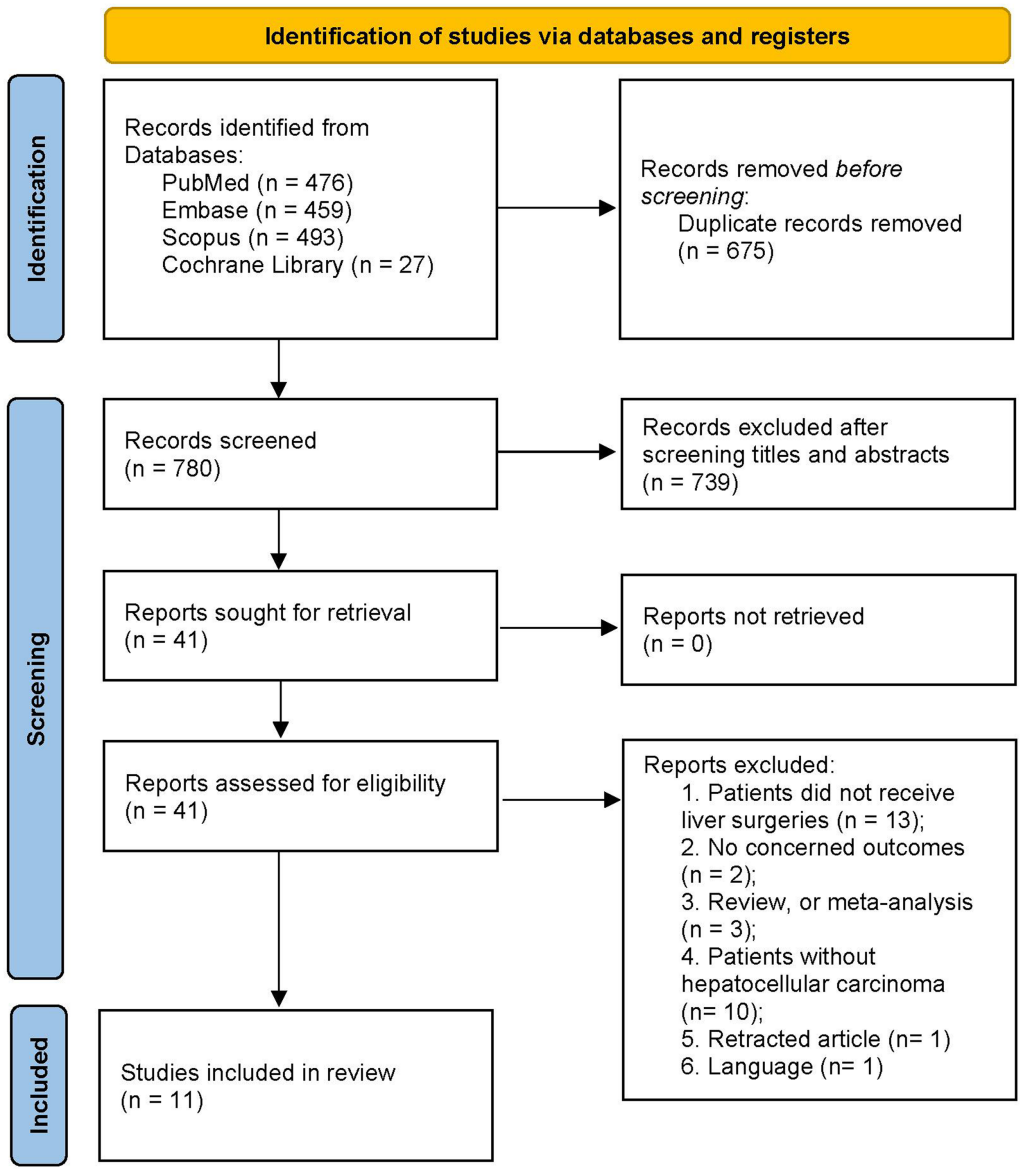
PRISMA 2020 flow diagram for the meta-analysis.

The initial search yielded a total of 1,455 articles from multiple sources: PubMed (*n* = 476), Embase (*n* = 459), Scopus (*n* = 493), and the Cochrane Library (*n* = 27). All records were imported into a document management software, from which 675 duplicated articles were automatically removed. Abstract screening resulted in the exclusion of 739 ineligible studies. Full-text evaluation led to the exclusion of 30 studies for various reasons (refer to Supplementary material 2). Finally, our meta-analysis included 11 studies (16, 17, 19, 26–33) encompassing 6,390 patients with HCC. Figure 1 illustrates the flow chart of the search and study selection processes.

Table 1 summarizes the basic characteristics of the included studies. The sample size ranged from 104 to 1,442 patients across the included studies. Most of included trials were conducted in Asian populations: five in Japan, four in China, one in Korea. One additional study was from Australia. Each study employed different CAR cutoff values, ranging from 0.027 up to 0.5. In addition, one study (28) reported the ALB to CRP ratio, which was converted to CAR for consistency.

**Table 1:** Characteristics of included studies.

Study, year and country	*N*	Population	Clinicopathological features	Cutoff value	Outcomes, follow-up period
Mai 2024, China	1,039	HCC patients underwent initial curative liver resection from September 2013 to June 2019	BCLC stage: 0/A (54.8%), B (20.9%), C (24.4); Positive HBsAg (84.5%); Cirrhosis (49.1%)	0.11	OS, DFS; 5 years
Aida 2024, Japan	214	HCC patients underwent primary hepatic resection between January 2008 to December 2018	Positive HBsAg (21.0%); Multiple tumors (20.0%); Microvascular invasion (16.0%)	0.028	OS, DFS; 5 years
Peri 2023, Australia	157	Patients underwent liver resection for HCC between March 2007 and December 2020	Cirrhosis (65.0%); Vascular invasion (36.2%); Lymphatic invasion (14.7%); Poorly differentiation (33.1%)	0.034	OS, DFS; 5 years
Matsumoto 2022, Japan	497	Patients underwent first hepatic resection for HCC from January 2000 to December 2019	Positive HBsAg (21.3%); Microvascular invasion (79.3%)	0.037	OS; 5 years
Haruki 2022, Japan	210	HCC patients underwent primary hepatic resection between January 2008 and December 2018	Positive HBsAg (21.9%); Poorly differentiation (13.8%); Multiple tumors (20.5%); Microvascular invasion (16.7%)	0.04	OS, DFS; 3 years
Yamamoto 2019, Japan	478	HCC patients underwent hepatectomy between January 2009 and December 2015	Positive HBsAg (15.8%); Multiple tumors (34.9%); Poorly differentiation (11.1%); Microvascular invasion (20.1%)	0.027	OS, DFS; 10 years
Wu 2019, China	409	HCC patients underwent hepatectomy between January 2008 to December 2012	Positive HBsAg (87.0%); TNM: I/II (56.5%), III/IV (43.5%)	0.185	OS, DFS; 5 years
Shimizu 2018, Japan	239	Patients with HCC received initial liver resection between April 2006 and December 2013	Positive HBsAg (12.1%); TNM: I/II (54.4%), III/IV (45.6%); Microvascular invasion (31.4%)	0.028	OS; 5 years
Ren 2018, China	187	HCC patients with radical operation as initial treated between June 2012 and May 2017	Positive HBsAg (79.7%); Cirrhosis (64.7%); Multiple tumors (10.2%); Microvascular invasion (17.6%)	0.037	OS, DFS; 5 years
Oh 2018, Korea	389	Patients with HCC and underwent curative resection from January 2004 to December 2013	Positive HBsAg (6.9%); Cirrhosis (40.1%)	0.5	OS, DFS; 5 years
Pang 2017, China	247	Patients with HCC and underwent curative liver resection from January 2007 to December 2014	Positive HBsAg (84.6%); Cirrhosis (77.3%); TNM: I/II (42.9%), III/IV (57.1%)	0.09	OS, DFS; 5 years

*N, number of patients; OS, overall survival; DFS, disease-free survival; HCC, hepatocellular carcinoma.*

All studies were assessed as high quality, with total scores exceeding 6 (Supplementary material 2). However, significant publication bias was detected for both OS and DFS outcomes (OS: *p* = 0.0039, DFS: *p* = 0.0115, Supplementary material 3). To address this bias, the trim-and-fill method was employed, resulting in adjusted pooled HRs of 1.76 (95%CI 1.55 to 2.01, I^2^ = 5%, Supplementary material 3) for OS and 1.74 (95%CI 1.55 to 1.95, I^2^ = 0%, Supplementary material 3) for DFS.

### Meta-analysis results

**Figure 2:**
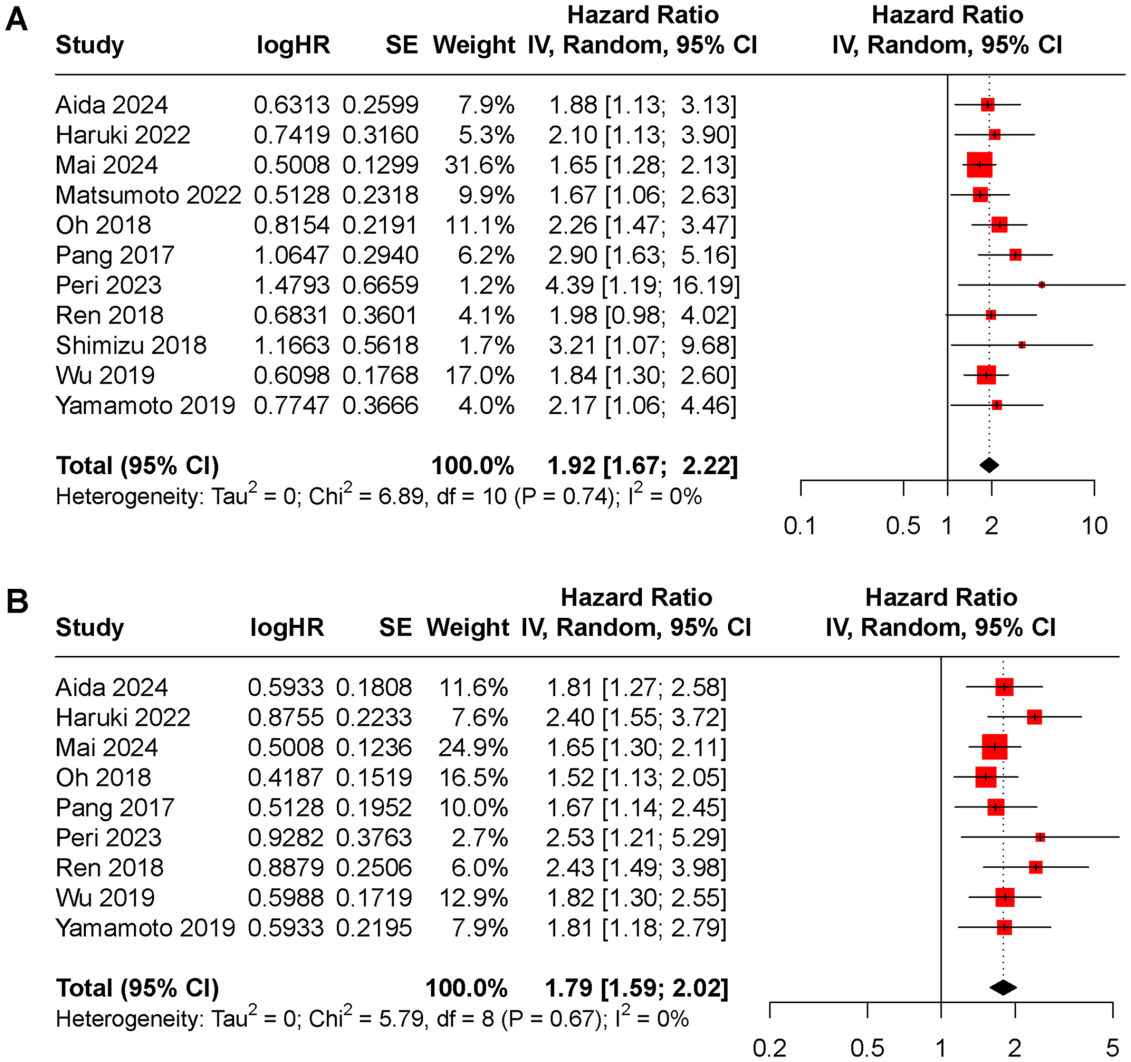
Forest plot of the pooled HR between low and high CAR groups for **(A)** OS rate, **(B)** DFS rate.

All included studies reported OS rate. The random-effects model indicated that higher preoperative CAR was associated with poorer OS (HR 1.92, 95% CI 1.67 to 2.22, *I*^2^ = 0%, Figure 2A). DFS rate was evaluated in 9 included studies, with a pooled HR of 1.79 (95% CI 1.59 to 2.02, *I*^2^ = 0%, Figure 2B) for higher versus lower CAR groups.

**Figure 3:**
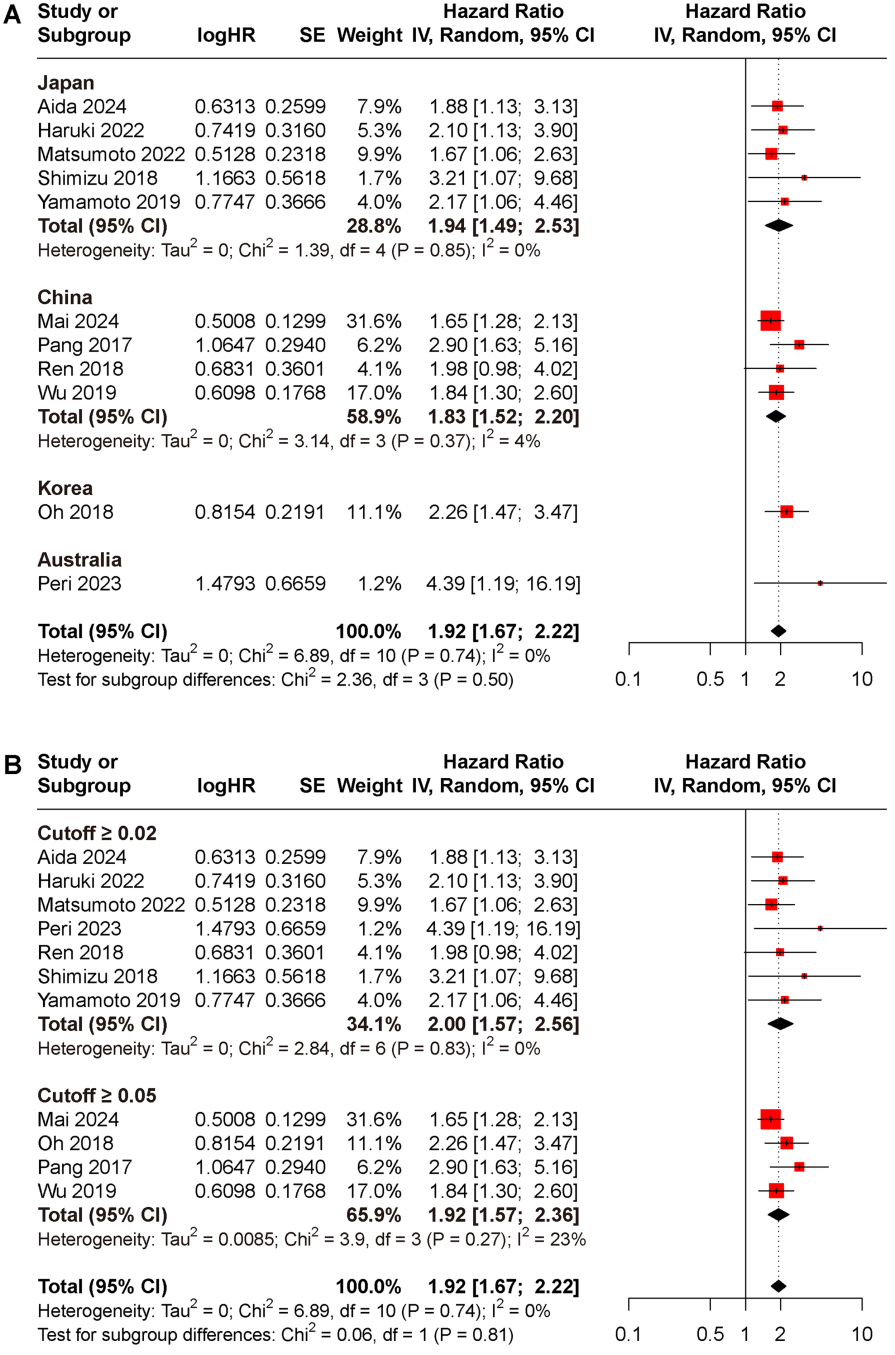
Subgroup analyses for OS rate between low and high CAR groups, **(A)** different reign; **(B)** cutoff ≥0.02 versus ≥0.05.

**Figure 4:**
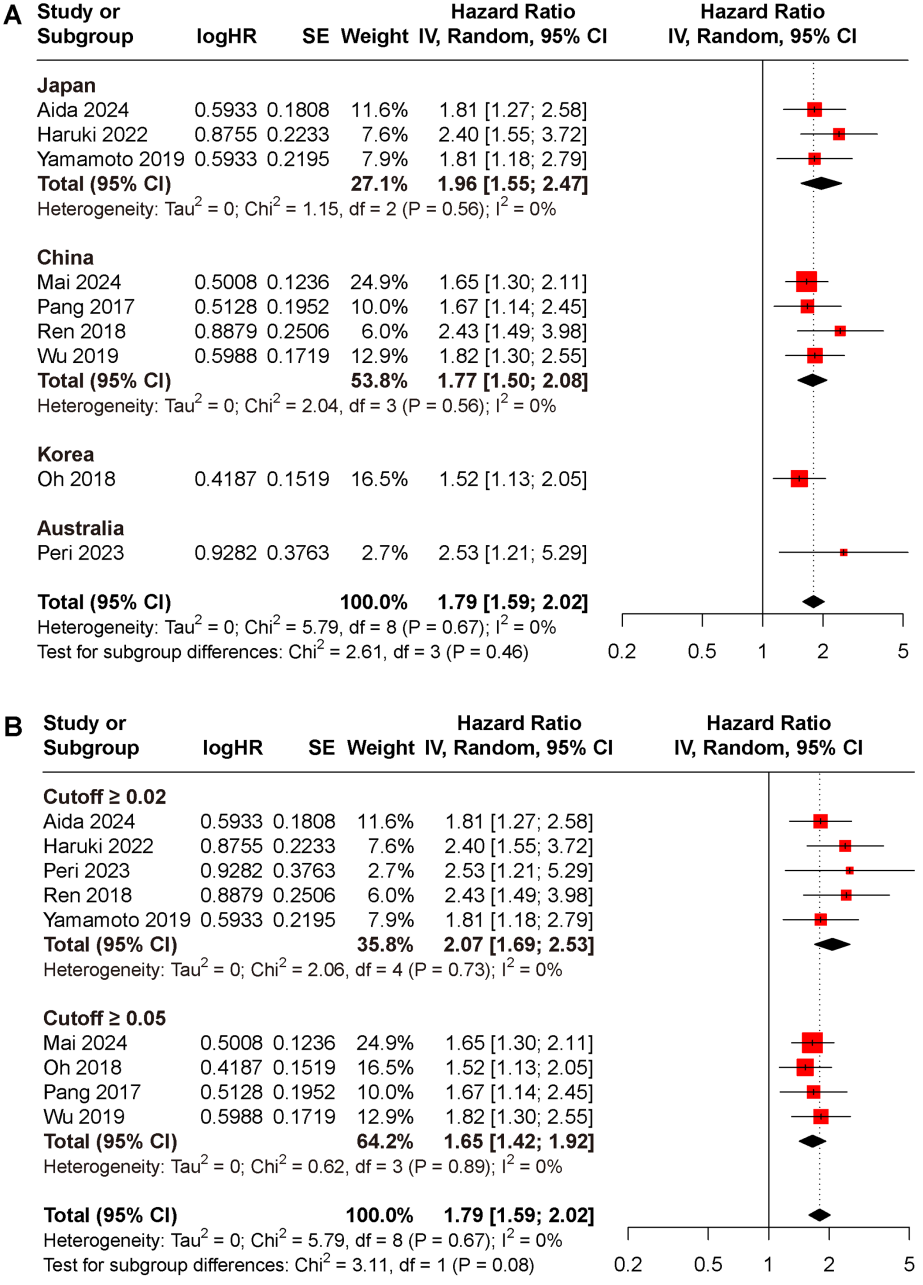
Subgroup analyses for DFS rate between low and high CAR groups, **(A)** different reign; **(B)** cutoff ≥0.02 versus ≥0.05.

Subgroup analyses for OS and DFS stratified by regions and cut-off value were conducted (Figures 3, 4). Results indicated that preoperative CAR could serve as a prognostic biomarker for patients with HCC across different regions (Japan: HR for OS 1.94, 95% CI 1.49 to 2.17, *I*^2^ = 71%, HR for DFS 1.96, 95% CI 1.55 to 2.47, *I*^2^ = 0%; China: HR for OS 1.83, 95% CI 1.52 to 2.20, *I*^2^ = 4%, HR for DFS 1.77, 95% CI 1.50 to 2.08, *I*^2^ = 0%; Korea: HR for OS 2.26, 95% CI 1.47 to 3.47, HR for DFS 1.52, 95% CI 1.13 to 2.05; Australia: HR for OS 4.39, 95% CI 1.19 to 16.19, HR for DFS 2.53, 95% CI 1.21 to 5.29) and cut-off values (cutoff ≥0.02: HR for OS 2.00, 95% CI 1.57 to 2.56, *I*^2^ = 0%, HR for DFS 2.07, 95% CI 1.69 to 2.53, *I*^2^ = 0%; cutoff ≥0.05: HR for OS 1.92, 95% CI 1.57 to 2.36, *I*^2^ = 23%, HR for DFS 1.65, 95% CI 1.42 to 1.92, *I*^2^ = 0%).

Sensitivity analyses showed robust pooled effect estimates (Supplementary material 3), with HRs ranging from 1.87 (95%CI 1.62 to 2.17) to 2.07 (95%CI 1.74 to 2.46) for OS, and 1.75 (95%CI 1.54 to 1.98) to 1.85 (95%CI 1.62 to 2.11) for DFS.

**Figure 5:**
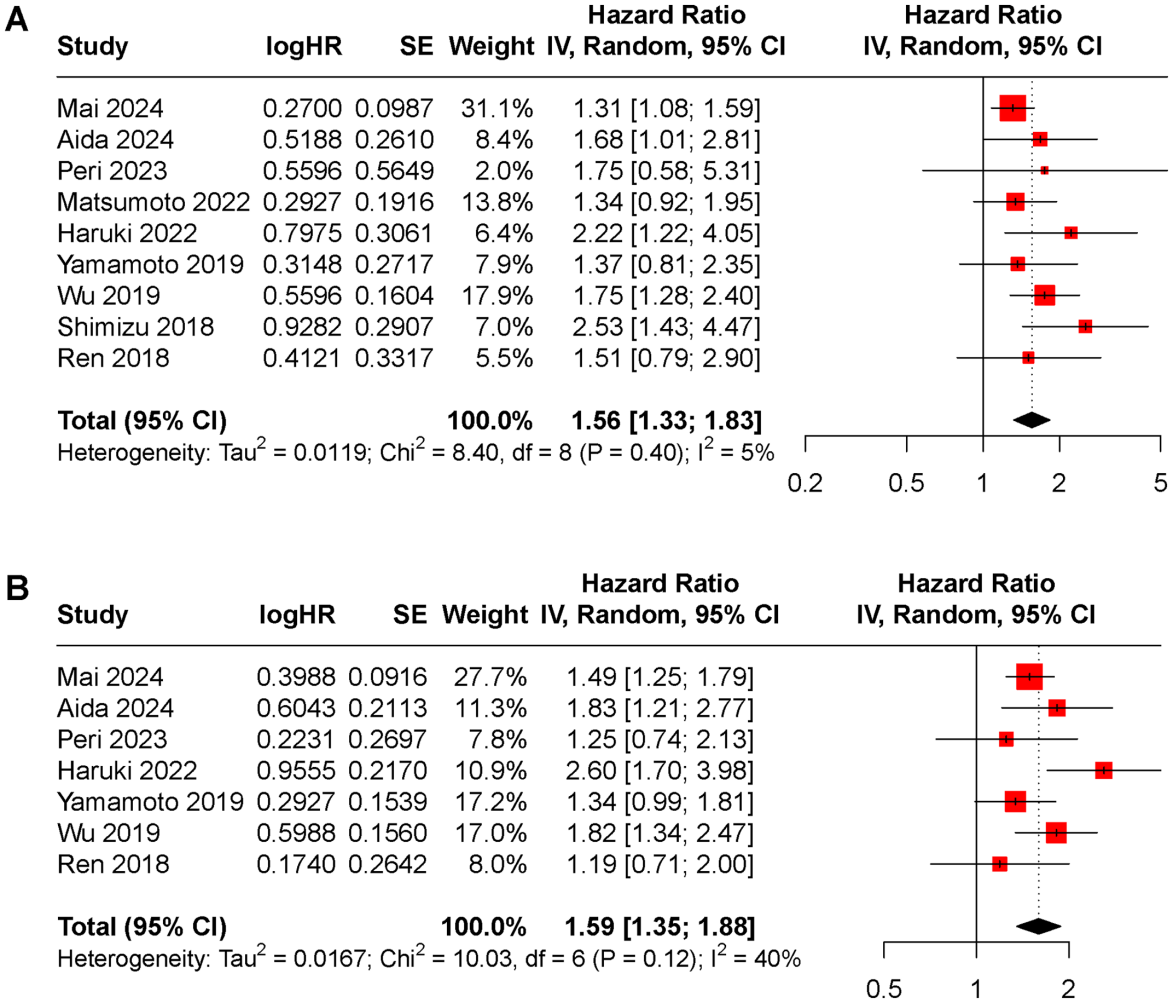
Forest plot of the pooled HR between low and high AFP groups for **(A)** OS rate, **(B)** DFS rate.

Furthermore, the prognostic value of CAR was compared with alpha-fetoprotein (AFP) using data from nine studies that reported HRs for AFP in relation to OS and DFS. The pooled HRs for AFP were significant for both OS (HR 1.56, 95% CI 1.33 to 1.83, *I*^2^ = 5%, Figure 5A) and DFS (HR 1.59, 95% CI 1.35 to 1.88, *I*^2^ = 40%, Figure 5B).

## Discussion

In this updated meta-analysis investigating the prognostic value of preoperative CAR, we found that elevated preoperative CAR was significantly associated with poor OS and DFS rate among patients undergoing hepatectomy for HCC. Patients with high preoperative CAR exhibited a 1.78-fold reduction in OS and a 1.63-fold reduction in DFS compared to those with low CAR levels. This association remained consistent across different reigns, suggesting that preoperative CAR demonstrates promising prognostic value in various subgroups. Notably, preoperative CAR showed a stronger association with poor prognosis compared to AFP. However, the lack of a unified standard for establishing cutoff values presents a challenge that future studies need to address.

The inflammatory tumor microenvironment is closely implicated in all stages of cancer development, including initiation, promotion, and progression (34–36). Current evidence recognizes systemic inflammation response as a crucial factor in promoting tumorigenesis and cancer progression, with particular relevance to adverse outcomes in patients with HCC (37). Numerous studies have highlighted the significance of inflammation-based scoring systems in predicting the prognosis of HCC (38, 39). These scoring systems, such as the neutrophil-to-lymphocyte ratio (NLR), monocyte-to-lymphocyte ratio (MLR), platelet-to-lymphocyte ratio (PLR), and systemic immune-inflammation index (SII), typically comprise acute phase inflammatory cells from the circulatory system. Among these, the NLR, a predictive model based on circulating leukocytes, has demonstrated satisfactory prognostic value in patients with HCC (40, 41). Other studies have also found that the PLR, MLR, as well as SII, are useful predictors of outcomes in HCC patients (42, 43). However, a notable proportion of HCC patients undergo adjuvant therapy prior to hepatectomy, including hepatic artery infusion chemotherapy, targeted therapy, and radiofrequency ablation. These treatments can lead to a reduction in circulating inflammatory cells such as neutrophils, lymphocytes, monocytes, and platelet (37). Consequently, the leukocyte-based models mentioned earlier may not accurately reflect the true systemic inflammatory response status of HCC patients. As a result, they may be inadequate for evaluating the prognosis of HCC patients who have undergone hepatectomy.

Nutritional status is a crucial indicator significantly correlated with adverse outcomes in many cancer patients. Poor nutritional status heightens susceptibility to infection, vascular fragility, impaired wound healing, and dysfunctional coagulation, thus escalating the risk of serious postoperative complications, even mortality (44). CRP and ALB are vital acute-phase proteins reflecting the body’s nutritional state during systemic inflammatory responses and are pivotal in predicting cancer patient survival. The Glasgow prognostic score (GPS) and modified GPS (mGPS), comprising these acute-phase proteins, are recognized as independent prognostic markers HCC patients (45, 46). However, these markers are categorical. Conversely, the C-reactive protein to albumin ratio (CAR), also based on serum CRP and ALB, has been associated with a poor prognosis in HCC patients (20, 21). Despite using the same factors as GPS and mGPS, CAR can meticulously and rigorously stratify patient outcomes due to its continuous properties, potentially outperforming the sole use of serum CRP and albumin levels for scoring purposes.

To the best of our knowledge, this study represents the most comprehensive meta-analysis evaluating the prognostic value of preoperative CAR for survival outcomes after hepatectomy. A total of 11 studies comprised 4,066 patients with HCC undergoing hepatectomy were analyzed. The results indicated that high preoperative CAR was independently associated with poor OS and DFS among patients with HCC. Subgroup analyses demonstrated robust performance across different geographical locations. Collectively, our findings align with and substantiate the outcomes documented in previous meta-analyses (20, 21), thereby strengthening the existing body of evidence. Fan et al. (20) analyzed seven retrospective studies and found that HCC patients with high pre-treatment CAR had 2.48-fold reduced OS and 1.90-fold reduced DFS rate compared to those with low CAR. However, not all included patients received hepatectomy, potentially introducing selection bias due to differences in condition and treatment. Moreover, one of the included studies only reported the CRP to lymphocyte ratio instead of CRP, which could not be included in meta-analysis. Subsequently, Lin et al. (21) investigated the prognostic significance of CAR in Asian populations by including seven studies in China, Japan, and Korea. They discovered that the CAR was strongly correlated with the prognosis of patients with HCC and could serve as a noninvasive prognostic biomarker in clinical settings.

However, mechanism underlying the prognostic value of CAR in HCC remains unclear. Potential explanations may include the following: (1) Many solid tumors exhibit a strong association with inflammation, with CRP being one of the most common systemic inflammatory markers. (2) Nutritional status is a critical factor in the long-term prognosis of cancer patients, garnering increasing attention. ALB level serves as a simple marker to assess nutritional status. (3) ALB is exclusively synthesized in the liver, potentially enhancing the prognostic significance of CAR in HCC compared to other solid tumors.

### Limitations

Nonetheless, this meta-analysis has several limitations that necessitate cautious interpretation of the findings. (1) The primary constraint of this meta-analysis pertains to the retrospective nature of all the included studies. This may introduce potential selection and recall biases, which could lead to an overestimation of the prognosis value of CAR. (2) Clinical heterogeneity is expected to contribute to statistical heterogeneity in the results. Despite efforts to account for this through subgroup analyses, confounding factors such as age, gender, liver function, and primary diseases may still affect the results. (3) Varying cut-off values for CAR across studies hindered the determination of an optimal threshold. This lack of standardization makes it challenging to establish a universally applicable prognostic value for CAR in clinical practice. (4) Despite comprehensive literature search efforts, the possibility of unpublished studies due to negative outcomes remains. This potential publication bias could skew the overall results toward a more positive association between CAR and prognosis. (5) Some incomplete data such as histopathological image after biopsy may have limited our ability to fully explore potential subgroup effects or moderating factors.

## Conclusion

This meta-analysis provides evidence that elevated preoperative CAR is a significant predictor of poor OS and DFS in patients undergoing hepatectomy for HCC. Our findings suggest that CAR may serve as a valuable prognostic biomarker in this patient population. However, given the retrospective nature of the analyzed studies and the lack of a standardized cutoff value, further research is needed to verify the current findings. Furthermore, future multicenter, larger sample, and prospective randomized trials should investigate whether the application of CAR for patients with HCC can improve survival outcomes.

## Data Availability

The original contributions presented in the study are included in the article/Supplementary material, further inquiries can be directed to the corresponding author.

## Footnotes

^1^ https://osf.io/uavt8
