# Supplementary material for: Preoperative C-reactive protein to albumin ratio may be a good prognostic marker in patients undergoing hepatectomy for hepatocellular carcinoma: a meta-analysis
Source: Front Nutr. 2024 Oct 1;11:1444352. doi: 10.3389/fnut.2024.1444352 (PMC11475710; doi:10.3389/fnut.2024.1444352)
Supplement: Supplementary file 2 [file Table_2.DOCX]

**Supplementary Material 2:** Searching strategies, list of excluded studies with reasons, quality assessment of included studies by the Newcastle-Ottawa Scale

Pubmed

#1 Liver Neoplasms [MeSH] OR Carcinoma, Hepatocellular [MeSH] OR liver cancer [Title/Abstract] OR hepatoma [Title/Abstract] OR Hepatic carcinoma [Title/Abstract] OR liver carcinoma [Title/Abstract] OR hepatocellular carcinoma [Title/Abstract] OR hepatic cancer [Title/Abstract] OR colorectal liver metastases [Title/Abstract]

#2 ((C-reactive protein [Title/Abstract] AND albumin [Title/Abstract] AND ratio [Title/Abstract]) OR (car [Title/Abstract]) OR (C-Reactive Protein-Albumin Ratio [Title/Abstract]))

#1 AND #2

Embase

#1 ‘Liver Neoplasms’:ab,ti OR ‘liver cancer’:ab,ti OR ‘Hepatic carcinoma’:ab,ti OR ‘hepatic cancer’:ab,ti OR ‘hepatocellular carcinoma’:ab,ti OR ‘liver carcinoma’:ab,ti OR ‘colorectal liver metastases’:ab,ti

#2 ‘car’:ab,ti OR ‘C-Reactive Protein-Albumin Ratio’:ab,ti

#1 AND #2

Scopus

#1 TITLE-ABS-KEY (Liver Neoplasms) OR TITLE-ABS-KEY (liver cancer) OR TITLE-ABS-KEY (Hepatic carcinoma) OR TITLE-ABS-KEY (hepatic cancer) OR TITLE-ABS-KEY (hepatocellular carcinoma) OR TITLE-ABS-KEY (liver carcinoma) OR TITLE-ABS-KEY (colorectal liver metastases)

#2 TITLE-ABS-KEY (C-Reactive Protein-Albumin Ratio) OR TITLE-ABS-KEY (car)

#1 AND #2

Cochrane Library

#1 ‘Liver Neoplasms’:ti,ab,kw OR ‘liver cancer’: ti,ab,kw OR ‘Hepatic carcinoma’: ti,ab,kw OR ‘hepatic cancer’: ti,ab,kw OR ‘hepatocellular carcinoma’: ti,ab,kw OR ‘colorectal liver metastases’: ti,ab,kw OR ‘liver carcinoma’: ti,ab,kw

#2 C-Reactive Protein-Albumin Ratio:ti,ab,kw OR ‘car’: ti,ab,kw

#1 AND #2

**list of excluded studies with reasons**

| **Author, year and reference** | **Reason for exclusion** |
| --- | --- |
| Li et al. (2024) [1] | Patients with HCC treated with PD-1 inhibitors, without liver surgeries |
| Sadagopan et al. (2024) [2] | Review |
| Sahakyan et al. (2023) [3] | Patients with CRLM, without HCC |
| Li et al. (2022) [4] | Patients with HCC treated with transcatheter chemoembolization, without liver surgeries |
| Tada et al. (2022) [5] | Patients with unresectable HCC treated with lenvatinib, without liver surgeries |
| Utsumi et al. (2022) [6] | Patients with CRLM, without HCC |
| Kanno et al. (2022) [7] | Patients with CRLM, without HCC |
| Teng et al. (2022) [8] | Patients with unresectable HCC treated with atezolizumab plus bevacizumab, without liver surgeries |
| Arakawa et al. (2021) [9] | Patients with pancreatic cancer, without HCC |
| Mei et al. (2021) [10] | Patients with HCC treated with PD-1 inhibitors, without liver surgeries |
| Deng et al. (2021) [11] | Patients with CRLM, without HCC |
| Xing et al. (2021) [12] | Review |
| Ahiko et al. (2021) [13] | Patients with colorectal cancer, without liver surgeries |
| Oikonomou et al. (2020) [14] | Patients with cirrhosis, without HCC |
| Chen et al. (2020) [15] | Patients with HCC treated with radiofrequency ablation, without liver surgeries |
| Sakamoto et al. (2020) [16] | Patients with CRLM, without HCC |
| Wu et al. (2020) [17] | Retracted article |
| Lin et al. (2020) [18] | Meta-analysis |
| Sprinzl et al. (2019) [19] | Patients with HCC treated with sorafenib, without liver surgeries |
| Shen et al. (2019) [20] | Patients with HCC treated with transcatheter arterial chemoembolization and radiofrequency ablation, without liver surgeries |
| Shibutani et al. (2019) [21] | Article did not report concerned outcomes |
| Chen et al. (2018) [22] | Not all patients with HCC treated with liver surgeries |
| Liao et al. (2018) [23] | C-reactive protein/albumin ratio was not reported in the article |
| Honda et al. (2016) [24] | Patients with HCC treated with radiofrequency ablation, without liver surgeries |
| Li et al. (2016) [25] | Article was not published in English |
| Ishizuka et al. (2016) [26] | Patients with colorectal cancer, without HCC |
| Solaini et al. (2016) [27] | Patients with CRLM, without HCC |
| Wang et al. (2016) [28] | Not all patients with HCC treated with liver surgeries |
| Liu et al. (2015) [29] | Patients with gastric cancer, without HCC |
| Kinoshita et al. (2015) [30] | Not all patients with HCC treated with liver surgeries |

**Reference**

1. Li BB, Chen LJ, Lu SL, Lei B, Yu GL, Yu SP: **C-reactive protein to albumin ratio predict responses to programmed cell death-1 inhibitors in hepatocellular carcinoma patients**. *World J Gastrointest Oncol* 2024, **16**(1):61-78.

2. Sadagopan N, He AR: **Recent Progress in Systemic Therapy for Advanced Hepatocellular Carcinoma**. *Int J Mol Sci* 2024, **25**(2).

3. Sahakyan MA, Brudvik KW, Angelsen JH, Dille-Amdam RG, Sandvik OM, Edwin B, Nymo LS, Lassen K: **Preoperative Inflammatory Markers in Liver Resection for Colorectal Liver Metastases: A National Registry-Based Study**. *World J Surg* 2023, **47**(9):2213-2220.

4. Li J, Yang S, Li Y, Li C, Xia Y, Zhu S, Xia J: **The C-Reactive Protein to Albumin Ratio Is an Independent Prognostic Factor in Patients with Hepatocellular Carcinoma Undergoing Transarterial Chemoembolization: A Large Cohort Study**. *Cardiovasc Intervent Radiol* 2022, **45**(9):1295-1303.

5. Tada T, Kumada T, Hiraoka A, Hirooka M, Kariyama K, Tani J, Atsukawa M, Takaguchi K, Itobayashi E, Fukunishi S *et al*: **C-reactive protein to albumin ratio predicts survival in patients with unresectable hepatocellular carcinoma treated with lenvatinib**. *Sci Rep* 2022, **12**(1):8421.

6. Utsumi M, Inagaki M, Kitada K, Tokunaga N, Kondo M, Yunoki K, Sakurai Y, Hamano R, Miyasou H, Tsunemitsu Y *et al*: **Lymphocyte-to-C-Reactive Protein Ratio Predicts Prognosis in Patients With Colorectal Liver Metastases Post-hepatic Resection: A Retrospective Study**. *Anticancer Res* 2022, **42**(10):4963-4971.

7. Kanno H, Hisaka T, Akiba J, Hashimoto K, Fujita F, Akagi Y: **C-reactive protein/albumin ratio and Glasgow prognostic score are associated with prognosis and infiltration of Foxp3+ or CD3+ lymphocytes in colorectal liver metastasis**. *BMC Cancer* 2022, **22**(1):839.

8. Teng W, Lin CC, Su CW, Lin PT, Hsieh YC, Chen WT, Ho MM, Wang CT, Chai PM, Hsieh JC *et al*: **Combination of CRAFITY score with Alpha-fetoprotein response predicts a favorable outcome of atezolizumab plus bevacizumab for unresectable hepatocellular carcinoma**. *Am J Cancer Res* 2022, **12**(4):1899-1911.

9. Arakawa Y, Miyazaki K, Yoshikawa M, Yamada S, Saito Y, Ikemoto T, Imura S, Morine Y, Shimada M: **Value of the CRP-albumin ratio in patients with resectable pancreatic cancer**. *J Med Invest* 2021, **68**(3.4):244-255.

10. Mei J, Sun XQ, Lin WP, Li SH, Lu LH, Zou JW, Wei W, Guo RP: **Comparison of the Prognostic Value of Inflammation-Based Scores in Patients with Hepatocellular Carcinoma After Anti-PD-1 Therapy**. *J Inflamm Res* 2021, **14**:3879-3890.

11. Deng Y, Zhao Y, Qin J, Huang X, Wu R, Zhou C, Pan Z: **Prognostic Value of the C-Reactive Protein/Albumin Ratio and Systemic Immune-Inflammation Index for Patients With Colorectal Liver Metastasis Undergoing Curative Resection**. *Pathol Oncol Res* 2021, **27**:633480.

12. Xing M, Wang X, Kiken RA, He L, Zhang JY: **Immunodiagnostic Biomarkers for Hepatocellular Carcinoma (HCC): The First Step in Detection and Treatment**. *Int J Mol Sci* 2021, **22**(11).

13. Ahiko Y, Shida D, Nakamura Y, Imaizumi J, Takamizawa Y, Moritani K, Tsukamoto S, Kanemitsu Y: **Preoperative Nutritional Scores as Host-Related Prognostic Factors for Both Overall Survival and Postoperative Complications in Patients With Stage II to III Colorectal Cancer**. *Dis Colon Rectum* 2021, **64**(10):1222-1231.

14. Oikonomou T, Goulis I, Kiapidou S, Tagkou N, Akriviadis E, Papatheodoridis G, Cholongitas E: **The significance of C-reactive protein to albumin ratio in patients with decompensated cirrhosis**. *Ann Gastroenterol* 2020, **33**(6):667-674.

15. Chen S, Ma W, Cao F, Shen L, Qi H, Xie L, Wu Y, Fan W: **Hepatocellular Carcinoma Within the Milan Criteria: A Novel Inflammation-Based Nomogram System to Assess the Outcomes of Ablation**. *Front Oncol* 2020, **10**:1764.

16. Sakamoto Y, Mima K, Imai K, Miyamoto Y, Tokunaga R, Akiyama T, Daitoku N, Hiyoshi Y, Iwatsuki M, Nagai Y *et al*: **Preoperative C-reactive protein-to-albumin ratio and clinical outcomes after resection of colorectal liver metastases**. *Surg Oncol* 2020, **35**:243-248.

17. Wu X, Sun Z, Zhu Y: **Prognostic effect of systemic inflammation in patients undergoing surgery for hepatocellular carcinoma: comparison of composite ratios and cumulative scores**. *Transl Cancer Res* 2020, **9**(10):6154-6165.

18. Lin N, Li J, Ke Q, Wang L, Cao Y, Liu J: **Clinical Significance of C-Reactive Protein to Albumin Ratio in Patients with Hepatocellular Carcinoma: A Meta-Analysis**. *Dis Markers* 2020, **2020**:4867974.

19. Sprinzl MF, Kirstein MM, Koch S, Seib ML, Weinmann-Menke J, Lang H, Düber C, Toenges G, Zöller D, Marquardt JU *et al*: **Improved Prediction of Survival by a Risk Factor-Integrating Inflammatory Score in Sorafenib-Treated Hepatocellular Carcinoma**. *Liver Cancer* 2019, **8**(5):387-402.

20. Shen Y, Wang H, Li W, Chen J: **Prognostic significance of the CRP/Alb and neutrophil to lymphocyte ratios in hepatocellular carcinoma patients undergoing TACE and RFA**. *J Clin Lab Anal* 2019, **33**(9):e22999.

21. Shibutani M, Nagahara H, Fukuoka T, Iseki Y, Hirakawa K, Ohira M: **Efficacy of Adjuvant Chemotherapy According to the Classification of Recurrence Risk Based on Systemic Inflammatory Markers in Patients With Liver Metastases of Colorectal Cancer**. *Anticancer Res* 2019, **39**(9):5039-5045.

22. Chen J, Fang A, Chen M, Tuoheti Y, Zhou Z, Xu L, Chen J, Pan Y, Wang J, Zhu H *et al*: **A novel inflammation-based nomogram system to predict survival of patients with hepatocellular carcinoma**. *Cancer Med* 2018, **7**(10):5027-5035.

23. Liao M, Chen P, Liao Y, Li J, Yao W, Sun T, Liao W, Su L: **Preoperative high-sensitivity C-reactive protein to lymphocyte ratio index plays a vital role in the prognosis of hepatocellular carcinoma after surgical resection**. *Onco Targets Ther* 2018, **11**:5591-5600.

24. Honda K, Seike M, Oribe J, Endo M, Arakawa M, Syo H, Iwao M, Tokoro M, Nishimura J, Mori T *et al*: **Risk factors for deterioration of long-term liver function after radiofrequency ablation therapy**. *World J Hepatol* 2016, **8**(13):597-604.

25. Man LI, Peng Z: **Predictive Value of C-reactive Protein/Albumin Ratio on Prognosis of Patients with Primary Hepatocellular Carcinoma**. *Cancer Research on Prevention and Treatment* 2016, **43**(07).

26. Ishizuka M, Nagata H, Takagi K, Iwasaki Y, Shibuya N, Kubota K: **Clinical Significance of the C-Reactive Protein to Albumin Ratio for Survival After Surgery for Colorectal Cancer**. *Ann Surg Oncol* 2016, **23**(3):900-907.

27. Solaini L, Atmaja BT, Arumugam P, Hutchins RR, Abraham AT, Bhattacharya S, Kocher HM: **The role of perioperative inflammatory-based prognostic systems in patients with colorectal liver metastases undergoing surgery. A cohort study**. *Int J Surg* 2016, **36**(Pt A):8-12.

28. Wang JQ, Cheng PG, Li MY, Ma LM: **Comparison of the prognostic value of C-reactive protein-based prognostic scores in patients with hepatitis B virus-related hepatocellular carcinoma**. 2016.

29. Liu X, Sun X, Liu J, Kong P, Chen S, Zhan Y, Xu D: **Preoperative C-Reactive Protein/Albumin Ratio Predicts Prognosis of Patients after Curative Resection for Gastric Cancer**. *Transl Oncol* 2015, **8**(4):339-345.

30. Kinoshita A, Onoda H, Imai N, Iwaku A, Oishi M, Tanaka K, Fushiya N, Koike K, Nishino H, Matsushima M: **The C-reactive protein/albumin ratio, a novel inflammation-based prognostic score, predicts outcomes in patients with hepatocellular carcinoma**. *Ann Surg Oncol* 2015, **22**(3):803-810.

**The quality assessment of included studies by the Newcastle-Ottawa Scale for cohort studies**

| Study | Newcastle-Ottawa Scale components | | | | | | | | Quality score |
| --- | --- | --- | --- | --- | --- | --- | --- | --- | --- |
|  | 1 | 2 | 3 | 4 | 5 | 6 | 7 | 8 |  |
| Mai 2024 | * | * | * | * |  | * | * | * | 7 |
| Aida 2024 | * | * | * | * | ** | * | * | * | 9 |
| Peri 2023 | * | * | * | * |  | * | * | * | 7 |
| Matsumoto 2022 | * | * | * | * |  | * | * | * | 7 |
| Haruki 2022 | * | * | * | * |  | * | * | * | 7 |
| Yamamoto 2019 | * | * | * | * |  | * | * | * | 7 |
| Wu 2019 | * | * | * | * |  | * | * | * | 7 |
| Shimizu 2018 | * | * | * | * |  | * | * | * | 7 |
| Ren 2018 | * | * | * | * |  | * | * | * | 7 |
| Oh 2018 | * | * | * | * |  | * | * | * | 7 |
| Pang 2017 | * | * | * | * |  | * | * | * | 7 |

1: Representativeness of the exposed cohort; 2: Selection of the non-exposed cohort; 3: Ascertainment of exposure; 4: Demonstration that outcome of interest was not present at start of study; 5: Comparability of cohorts on the basis of the design or analysis; 6: Assessment of outcome; 7: Was follow-up long enough for outcomes to occur; 8: Adequacy of follow up of cohorts.
