# Supplementary material for: Preoperative C-reactive protein to albumin ratio may be a good prognostic marker in patients undergoing hepatectomy for hepatocellular carcinoma: a meta-analysis
Source: Front Nutr. 2024 Oct 1;11:1444352. doi: 10.3389/fnut.2024.1444352 (PMC11475710; doi:10.3389/fnut.2024.1444352)
Supplement: Supplementary file 3 [file Image_1.pdf]

Supplementary Material 3: Publication bias assessment by funnel plot and Egger's test, sensitivity analyses

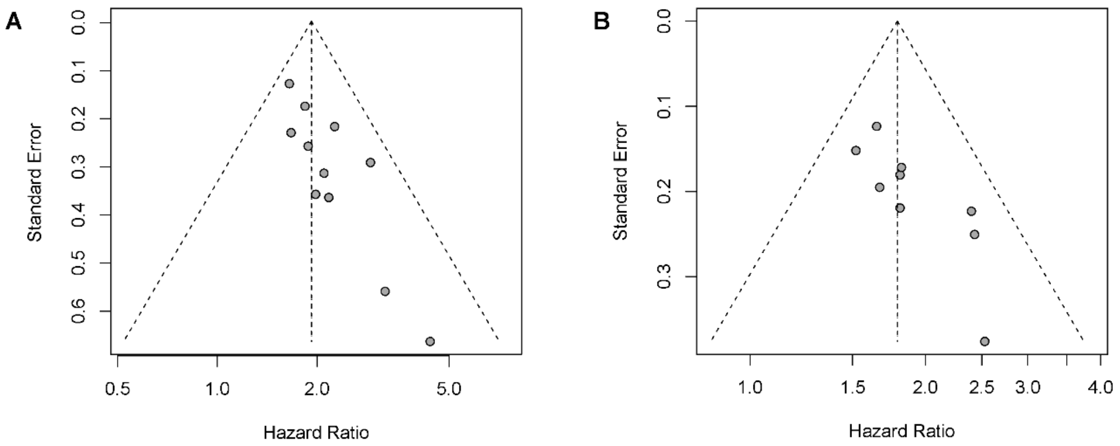

Figure 1: Funnel plot for (A) overall survival, Egger's test P = 0.0039, (B) disease free survival, Egger's test P = 0.0115

Study	logHR	SE	Weight	Hazard Ratio IV, Random, 95% CI
Aida 2024	0.6313	0.2599	6.4%	1.88 [1.13; 3.13]
Haruki 2022	0.7419	0.3160	4.3%	2.10 [1.13; 3.90]
Mai 2024	0.5008	0.1299	25.4%	1.65 [1.28; 2.13]
Matsumoto 2022	0.5128	0.2318	8.0%	1.67 [1.06; 2.63]
Oh 2018	0.8154	0.2191	8.9%	2.26 [1.47; 3.47]
Pang 2017	1.0647	0.2940	5.0%	2.90 [1.63; 5.16]
Peri 2023	1.4793	0.6659	1.0%	4.39 [1.19; 16.19]
Ren 2018	0.6831	0.3601	3.3%	1.98 [0.98; 4.01]
Shimizu 2018	1.1663	0.5618	1.4%	3.21 [1.07; 9.65]
Wu 2019	0.6098	0.1768	13.7%	1.84 [1.30; 2.60]
Yamamoto 2019	0.7747	0.3666	3.2%	2.17 [1.06; 4.45]
Filled: Yamamoto 2019	0.3598	0.3666	3.2%	1.43 [0.70; 2.94]
Filled: Oh 2018	0.3191	0.2191	8.9%	1.38 [0.90; 2.11]
Filled: Pang 2017	0.0698	0.2940	5.0%	1.07 [0.60; 1.91]
Filled: Shimizu 2018	−0.0318	0.5618	1.4%	0.97 [0.32; 2.91]
Filled: Peri 2023	−0.3448	0.6659	1.0%	0.71 [0.19; 2.61]
**Total (95% CI)**			**100.0%**	**1.76 [1.55; 2.01]**

Heterogeneity: $Tau^2 < 0.0001$; $Chi^2 = 15.80$, df = 15 (P = 0.40); $I^2 = 5\%$

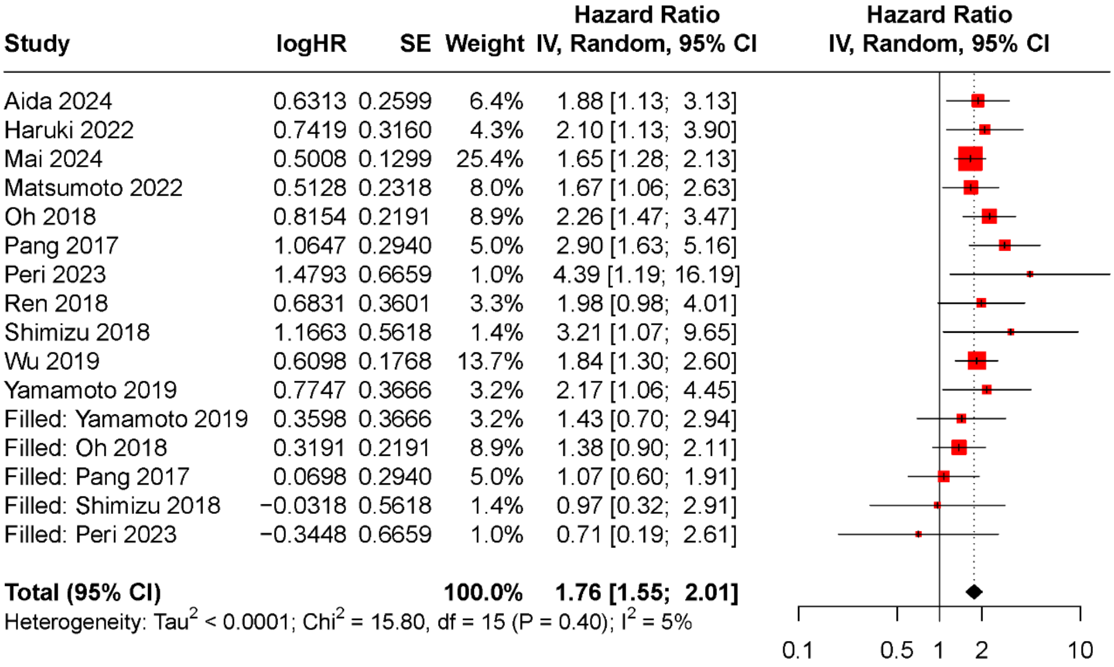

Figure 2: Forest plot for overall survival after trim-and-fill method

Study	logHR	SE	Weight	Hazard Ratio IV, Random, 95% CI
Aida 2024	0.5933	0.1808	10.7%	1.81 [1.27; 2.58]
Haruki 2022	0.8755	0.2233	7.0%	2.40 [1.55; 3.72]
Mai 2024	0.5008	0.1236	22.9%	1.65 [1.30; 2.10]
Oh 2018	0.4187	0.1519	15.1%	1.52 [1.13; 2.05]
Pang 2017	0.5128	0.1952	9.2%	1.67 [1.14; 2.45]
Peri 2023	0.9282	0.3763	2.5%	2.53 [1.21; 5.29]
Ren 2018	0.8879	0.2506	5.6%	2.43 [1.49; 3.97]
Wu 2019	0.5988	0.1719	11.8%	1.82 [1.30; 2.55]
Yamamoto 2019	0.5933	0.2195	7.2%	1.81 [1.18; 2.78]
Filled: Ren 2018	0.2164	0.2506	5.6%	1.24 [0.76; 2.03]
Filled: Peri 2023	0.1761	0.3763	2.5%	1.19 [0.57; 2.49]
**Total (95% CI)**			**100.0%**	**1.74 [1.55; 1.95]**

Heterogeneity: $Tau^2 < 0.0001$; $Chi^2 = 8.83$, df = 10 (P = 0.55); $I^2 = 0\%$

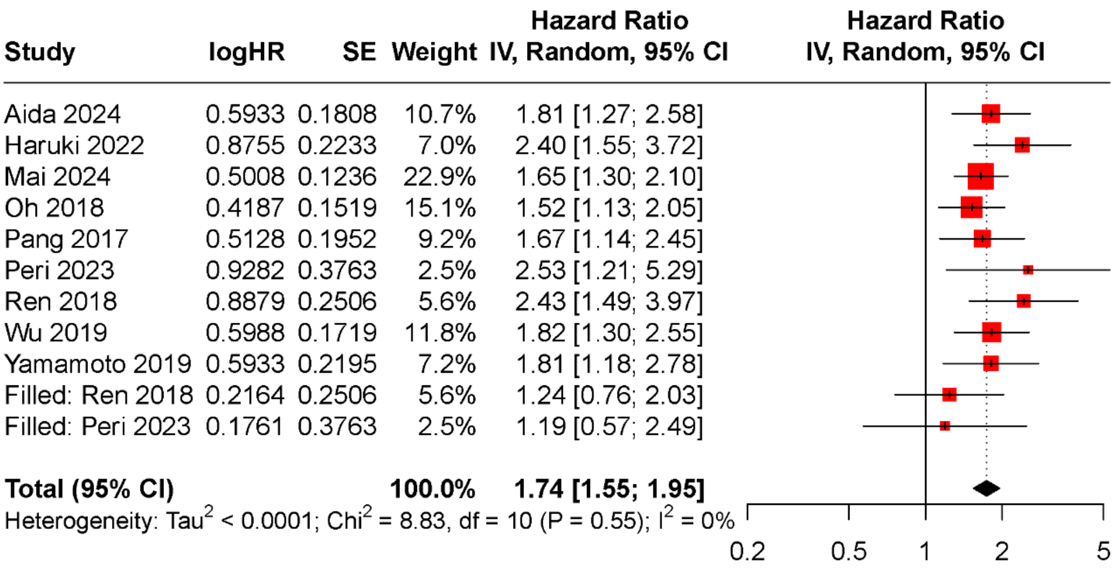

Figure 3: Forest plot for disease free survival after trim-and-fill method

Study	Hazard Ratio IV, Random, 95% CI
Omitting Aida 2024	1.93 [1.66; 2.24]
Omitting Haruki 2022	1.92 [1.65; 2.22]
Omitting Mai 2024	2.07 [1.74; 2.46]
Omitting Matsumoto 2022	1.96 [1.68; 2.29]
Omitting Oh 2018	1.89 [1.62; 2.20]
Omitting Pang 2017	1.87 [1.62; 2.17]
Omitting Peri 2023	1.91 [1.65; 2.20]
Omitting Ren 2018	1.92 [1.66; 2.22]
Omitting Shimizu 2018	1.91 [1.65; 2.20]
Omitting Wu 2019	1.96 [1.66; 2.31]
Omitting Yamamoto 2019	1.92 [1.66; 2.22]
**Total (95% CI)**	**1.92 [1.67; 2.22]**

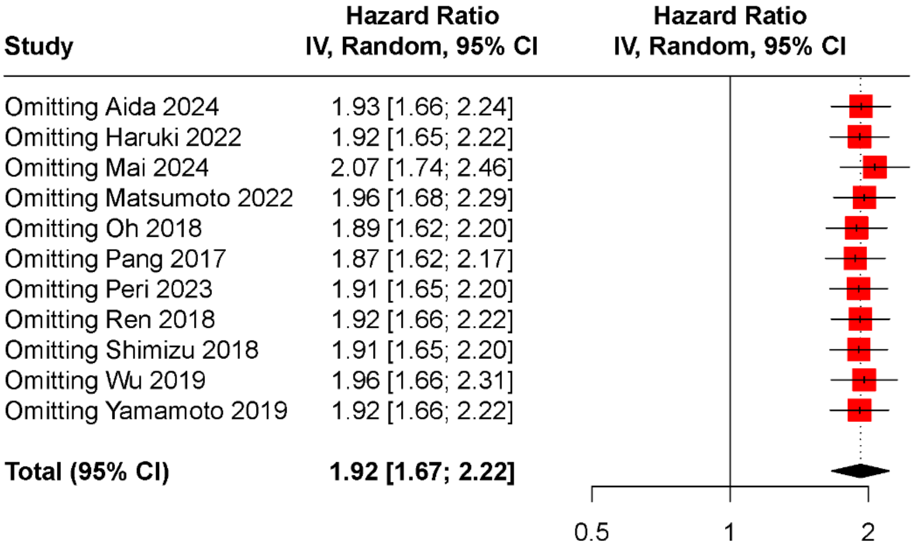

Figure 4: Sensitivity analysis for overall survival

Study	Hazard Ratio IV, Random, 95% CI
Omitting Aida 2024	1.79 [1.57; 2.03]
Omitting Haruki 2022	1.75 [1.54; 1.98]
Omitting Mai 2024	1.84 [1.60; 2.11]
Omitting Oh 2018	1.85 [1.62; 2.11]
Omitting Pang 2017	1.80 [1.59; 2.05]
Omitting Peri 2023	1.77 [1.57; 2.00]
Omitting Ren 2018	1.76 [1.55; 1.99]
Omitting Wu 2019	1.79 [1.57; 2.03]
Omitting Yamamoto 2019	1.79 [1.58; 2.03]
**Total (95% CI)**	**1.79 [1.59; 2.02]**

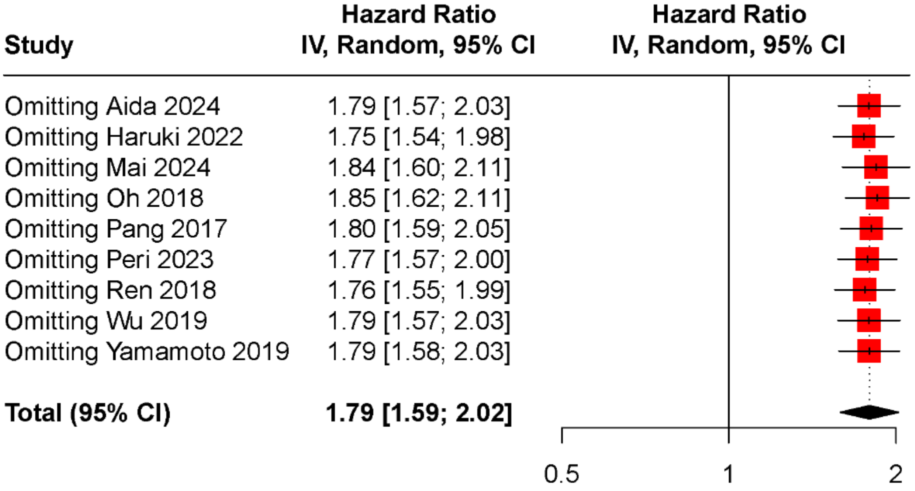

Figure 5: Sensitivity analysis for disease free survival
